# Molecular and morphological investigations of two new species in *Qinia* and *Cymbella* (Bacillariophyceae: Cymbellales) from China

**DOI:** 10.1371/journal.pone.0314880

**Published:** 2024-12-13

**Authors:** Jingshen Li, Andrei Mironov, Yutong Jiang, Jinyan Liang, John P. Kociolek, Yevhen Maltsev, Yawen Fan, Yan Liu, Maxim Kulikovskiy

**Affiliations:** 1 College of Life Science and Technology, Harbin Normal University, Harbin, China; 2 K.A. Timiryazev Institute of Plant Physiology RAS, IPP RAS, Moscow, Russia; 3 Faculty of Biology, M.V. Lomonosov Moscow State University, Moscow, Russia; 4 Museum of Natural History and Department of Ecology and Evolutionary Biology, University of Colorado, Boulder, Colorado, United States of America; CEA lRlG: Commissariat a l’energie atomique et aux energies alternatives lnstitut de Recherche Interdisciplinaire de Grenoble, FRANCE

## Abstract

Molecular data is provided firstly for the newly erected genus *Qinia*, and the phylogenetic position of the genus *Qinia* within the Cymbellales is discussed. Despite the presence of apical pore fields bisected by the distal raphe fissure being a very distinctive feature for *Qinia*, molecular analysis demonstrates this character as homoplasious, having evolved independently in *Qinia* and *Cymbella*. Two new species, *Qinia hubeii* sp. nov. and *Cymbella wuhanensis* sp. nov., are described based on multigene molecular investigation (genetic markers *18S rDNA*, *28S rDNA* and *rbcL*) and morphological comparisons with the diatoms from the family Cymbellaceae. Molecular data suggest a close relationship between *Qinia hubeii* sp. nov. and *Karthickia* and *Encyonopsis*, while *Cymbella wuhanensis* sp. nov. forms a clade with *Cymbella aspera* and *Cymbella bengalensis*. Morphological features of the new species were observed with light and scanning electron microscopy. Comparison of *Qinia hubeii* sp. nov. with other species in *Qinia* and *Cymbella wuhanensis* sp. nov. with similar *Cymbella* species were discussed.

## Introduction

Diatoms of the family Cymbellaceae Kützing comprise a diverse group of freshwater algae. The family is currently represented by 26 genera and more than a thousand species [1]. Since its original description [2], the classification of Cymbellaceae has been significantly modified. Krammer [3,4], who carried out a revision of *Cymbella* Agardh, determined the most taxonomically important features of the valve in this catch-all genus. More recently, new genera within the family Cymbellaceae were either split off from *Cymbella*, e.g. *Cymbellopsis* Krammer, *Afrocymbella* Krammer, *Gomphocymbellopsis* Krammer, *Delicatophycus* M.J. Wynne, *Celebesia* Kapustin, Kulikovskiy & Kociolek and *Alveocymba* Kapustin, Kulikovskiy & Kociolek [5–9] or discovered as new to science–e.g. *Oricymba* Jüttner, Krammer, Cox, Van de Vijver & Tuji, *Karthickia* Kociolek, Glushchenko & Kulikovskiy, *Vladinikolaevia* Kulikovskiy, Glushchenko, Y. Liu & Kociolek [10–12]. The distinguishable features among this group are symmetry of valve, location of the raphe, direction of the distal raphe ends, presence or absence of stigmata and apical pore fields (APFs) [4,13]. Recently, based on detailed light and scanning electron microscopy (LM and SEM) observations, more fine structures of the valve were found, including areolar occlusions, which provide more insights on the classification of this group. *Qinia* Y. Liu, Kociolek & Kulikovskiy, for instance, is a genus, recently discovered in China [13]. It resembles *Cymbella* in possessing dorsiventral valves, as well as the orientation of the raphe fissures and presence of the apical pore fields at both apices. However, *Qinia* exhibits some unique features seen with both LM and SEM: the valves are astigmate, areolar openings are slit-like to C-shaped and occluded internally with unilateral foricula (sensu Cox [14]), APFs are bisected by the distal raphe fissures [13]. Since the genus has been formally described only relatively recently, there is still limited information available about its phylogeny. Thus, molecular data is still wanting to clear the relationship with the cymbelloid genera. During our investigation of freshwater diatom flora in Hubei Province, China, two unknown species were found. One of the species found possesses the distinct morphological features of the genus *Qinia*, e.g. APFs bisected by fissures of the raphe and C-shaped openings of areolae. Another species belongs to the *Cymbella proxima* Reimer-group based on valve outline, position and structure of stigmata and APFs. Three strains were isolated from the collected samples and later used for molecular analysis of genetic markers *18S rDNA*, *28S rDNA* and *rbcL*. Our morphological and molecular data support that both species are new to science. Hence, we describe *Qinia hubeii* Y. Liu & Kociolek sp. nov. and *Cymbella wuhanensis* Y. Liu & Kociolek sp. nov. in this paper. For the first time, molecular data is acquired for *Qinia*. Molecular phylogenetic trees are built for 19 genera from the order Cymbellales D.G. Mann and related taxa, and the phylogenetic relationships between them are discussed.

## Materials and methods

### Sampling and culturing

Benthic samples, used in the present report were collected from Hubei, China, during two different expeditions to Jiufeng reservoir (Hubei, China) on 17 Sep. 2022 and 7 Oct. 2022 (Table 1). Three strains (CBac2022123, CBac2022127 and CBac2022142) were isolated from these samples. A subsample of each collection was added to CSI liquid medium. Strains were established by micropipetting single cells under an inverted microscope. Non-axenic strains were maintained in CSI liquid medium at 22–25°C with an alternating 12-hour light and dark photoperiod.

**Table 1:** Information about the studied samples.

Sample number	Strain number	Location and sample type	Geographical coordinates	Water parameters	Date of collection
THHB2022468	CBac2022123	Jiufeng reservoir, Hubei, China	30°29′44.16″ N, 114°33′21.60″ E	pH 8.0; 22.3°C; 350 μS/cm	17 Sep. 2022
THHB2022472	CBac2022127	Jiufeng reservoir, Hubei, China	30°29′44.16″ N, 114°33′21.60″ E	pH 8.0; 22.3°C; 350 μS/cm	17 Sep. 2022
THHB2022487	CBac2022142	Jiufeng reservoir, Hubei, China	30°29′42.00″ N, 114°33′24.84″ E	pH 8.3; 21.8°C; 378 μS/cm	07 Oct. 2022

### DNA extraction, PCR amplification and sequencing

**Fig 1:**
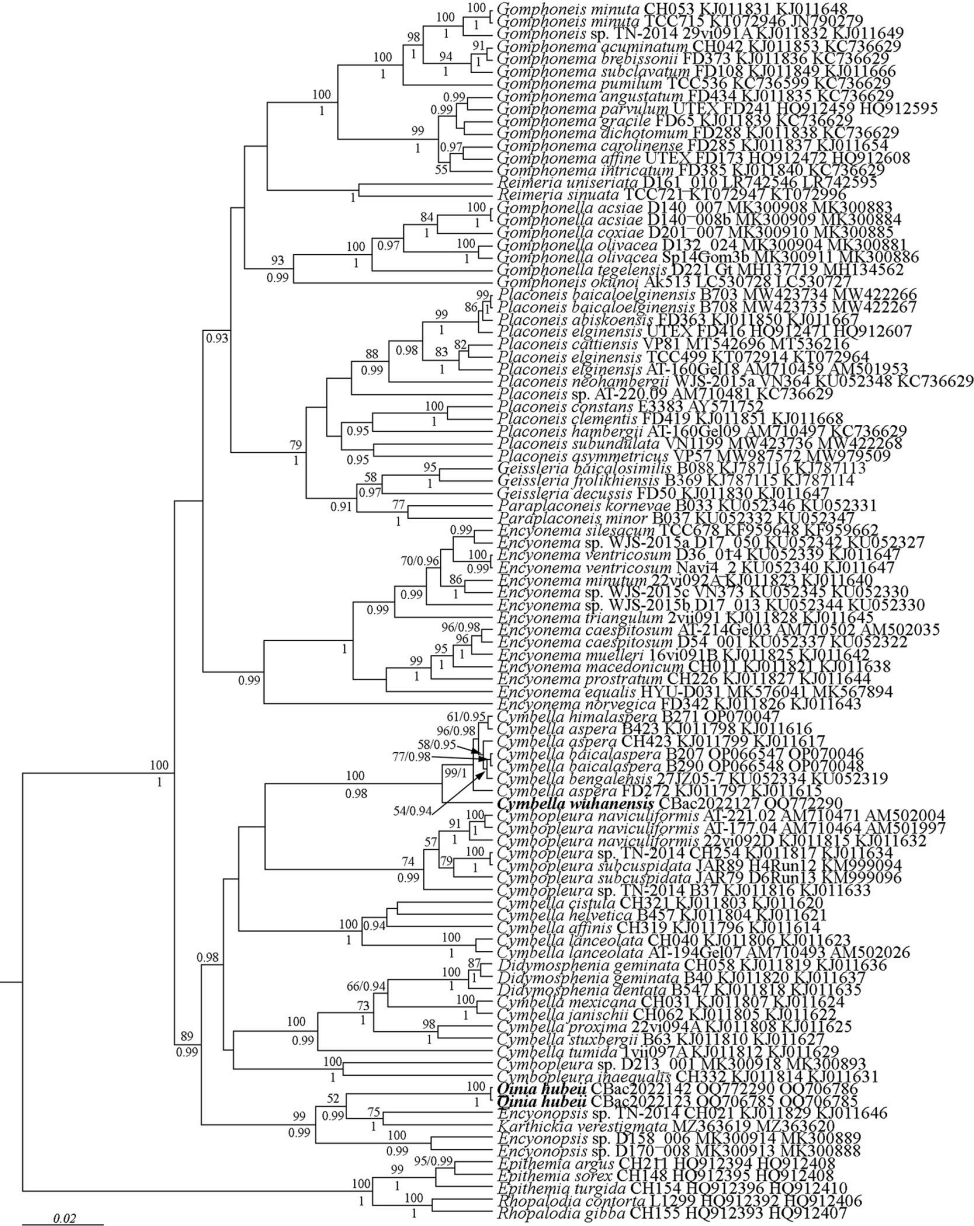
Phylogenetic position of *Cymbella wuhanensis* sp. nov. and *Qinia hubeii* sp. nov. (indicated in bold) based on Bayesian inference for the partial *18S rDNA* and *rbcL* genes. The total length of the alignment is 1706 characters. Bootstrap supports of ML and posterior probabilities of BI are presented on the nodes. Only likelihood bootstraps and posterior probabilities above 50 and 0.9 are shown. Strain numbers (if available) and GenBank numbers are indicated for all sequences.

**Fig 2:**
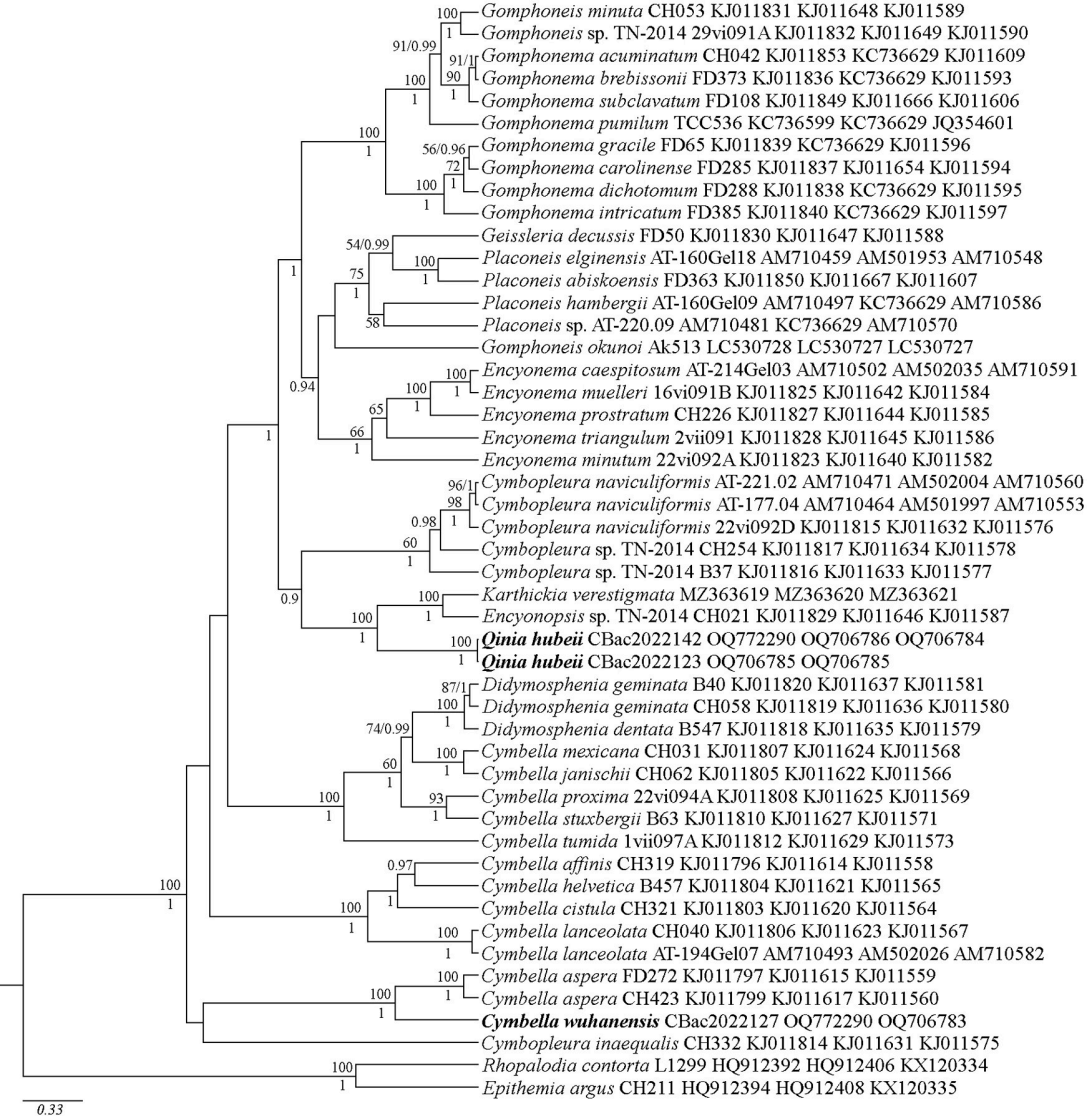
Phylogenetic position of *Cymbella wuhanensis* sp. nov. and *Qinia hubeii* sp. nov. (indicated in bold) based on Bayesian inference for the partial *18S rDNA*, *28S rDNA* and *rbcL* genes. The total length of the alignment is 2,273 characters. Bootstrap supports of ML and posterior probabilities of BI are presented on the nodes. Only likelihood bootstraps and posterior probabilities above 50 and 0.9 are shown. Strain numbers (if available) and GenBank numbers are indicated for all sequences.

Total DNA was extracted using Ezup columnar plant genomic DNA extraction kit (Shanghai Biotechnology Co., Ltd., China). Fragments of *18S rDNA* (600–604 bp, including V4 domain) were amplified using primers from Guo et al. [15]; *28S rDNA* (796–827 bp)–using primers DIR from Scholin et al. [16] and D3B from Nunn et al. [17]; partial *rbcL* plastid gene (867–1,101 bp)–using primers from Alverson et al. [18]. Amplification was carried out using premade polymerase chain reaction (PCR) premix (TaKaRa TaqTM Version 2.0 plus dye by TaKaRa, Japan). PCR products were visualized by horizontal electrophoresis in 1.0% agarose gel stained with GelStain (TransGen Biotech, China). The purified PCR products were sequenced by Sanger Sequencing method using a Genetic Analyzer 3500 instrument (Applied Biosystems, Waltham, MA, USA). The obtained sequences were edited manually and assembled using Ridom TraceEdit ver. 1.1.0 (Ridom GmbH, Münster, Germany) and Mega ver. 7 software [19]. Newly determined sequences and DNA fragments from 95 other diatoms, which were downloaded from GenBank (taxa and accession numbers are given in the trees, Figs 1 and 2), were included in the alignments. *Epithemia* Kützing and *Rhopalodia* Müller species were chosen as the outgroup taxa. The nucleotide sequences of the *18S rDNA*, *28S rDNA* and *rbcL* genes were aligned separately using the Mafft ver. 7 software and the E-INS-i algorithm [20]. A final alignment was then carried out: the resulting matrices were trimmed from the beginning and at the end, where nucleotide sites for the target sequences were unavailable. For the protein-coding sequences of the *rbcL* gene, we checked that the beginning of the aligned matrix corresponded to the first position of the codon (triplet). The resulting alignments had lengths of 611 (*18S rDNA*), 567 (*28S rDNA*) and 1,095 (*rbcL*) characters. Genetic distances (uncorrected pair-wise p-distance) were calculated by MEGA ver. X [21]. For each of the alignment partitions, the most appropriate substitution model, shape parameter α and a proportion of invariable sites (pinvars) were estimated using the Bayesian information criterion (BIC) as implemented in jModelTest ver. 2.1.10 [22]. Phylogenetic analyses were performed using the Maximum Likelihood (ML) and Bayesian Inference (BI) methods. This BI-criterion (BIC) based model selection procedure used the following models, shape parameter α and a proportion of invariable sites (pinvar): TrN+I+G, α = 0.4960 and pinvar = 0.4820 for *18S rDNA* in the two genes tree; TrN+I+G, α = 0.5660 and pinvar = 0.4930 for *18S rDNA* in the three genes tree; GTR+I+G, α = 0.6400 and pinvar = 0.2090 for *28S rDNA*; TVM+I+G, α = 0.4120, and pinvar = 0.6990 for the first codon position of the *rbcL* gene in the two genes tree; JC+I, pinvar = 0.8570 for the second codon position of the *rbcL* gene in the two genes tree; TVM+I+G, α = 1.1300, and pinvar = 0.2270 for the third codon position of the *rbcL* gene in the two genes tree; TVM+I+G, α = 0.5420, and pinvar = 0.5770 for the first codon position of the *rbcL* gene in the three genes tree; JC+I+G, α = 0.3440, and pinvar = 0.7480 for the second codon position of the *rbcL* gene in the three genes tree; TrN+I, pinvar = 0.4720 for the third codon position of the *rbcL* gene in the three genes tree. However, the F81 model was applied instead of JC, the GTR applied instead of TVM, TrN as the most similar applicable options for BI. Maximum Likelihood analysis was performed in IQ-TREE 2 [23], using the option of heuristic tree-searching with tree bisection-reconnection branch swapping. The BI was run for 5 million generations in Beast ver. 1.10.1 software [24]. Five MCMC analyses were run for 5 million generations (burn-in 1,000 million generations). The convergence diagnostics was performed in the Tracer ver. 1.7.1 software [24]. The initial 15% trees were removed, the rest retained to construct a final chronogram with 90% posterior probabilities. The median node height for each of the clades were used in the summary trees. The software FigTree ver. 1.4.3 [24] was used for viewing and editing of the trees.

## Results

### Molecular investigation

The phylogenetic analyses were conducted using three gene datasets (*18S rDNA*, *28S rDNA* and *rbcL*) and the results of two different analyses are shown in Fig 1 (based on fewer nucleotide sequence data but with more taxa) and Fig 2 (based on longer sequences but including fewer taxa). In Fig 1, the Cymbellales are shown to be monophyletic, but the Cymbellaceae are not monophyletic. One branch of the Cymbellales includes multiple species of the genera *Cymbella*, *Cymbopleura* (Krammer) Krammer, *Didymosphenia* M. Schmidt and *Encyonopsis* Krammer, as well as a single species each for *Karthickia* and *Qinia*. Of the genera included is this monophyletic lineage, only *Didymosphenia* is demonstrated to be monophyletic. All the other genera with multiple species included are non-monophyletic. Moreover, there is low support about the relationships of the major groupings within the Cymbellaceae. A second major branch of the Cymbellales includes *Encyonema* Kützing, *Paraplaconeis* Kulikovskiy, Lange-Bertalot & Metzeltin, *Geissleria* Lange-Bertalot & Metzeltin, *Placoneis* Mereschkowsky, *Gomphonella* Rabenhorst, *Gomphadelpha* R. Jahn & N. Abarca, *Reimeria* Kociolek & Stoermer and *Gomphonema* Ehrenberg. In this lineage, all genera are monophyletic except *Gomphonema*. However, higher level relationships of the major lineages within this group, especially within the genera traditionally considered part of the Gomphonemataceae Kützing (those with asymmetry about the transapical axis), lack strong statistical support. In Fig 2, which has fewer genera represented, monophyly of genera mirrors what was seen in Fig 1, with *Cymbella*, and *Cymbopleura* being non-monophyletic and *Didymosphenia* being monophyletic. In the second major lineage, only *Encyonema* and *Gomphadelpha* are monophyletic while the higher-level relationships within this clade do have high statistical support.

According to the ML and BI phylogenetic analyses (Figs 1 and 2), the three investigated strains, namely, CBac2022123 and CBac2022142 (representing *Qinia hubeii* sp. nov.), along with CBac2022127 (representing *Cymbella wuhanensis* sp. nov.) illustrate the overall phylogenies of *Qinia* and *Cymbella* with high statistical support (Figs 1 and 2). *Qinia hubeii* sp. nov. (strains CBac2022123 and CBac2022142) appeared most closely related to *Encyonopsis* strains and *Karthickia verestigmata* Glushchenko, Kulikovskiy & Kociolek and received high statistical support (Fig 1: ML 99; BI 0.99). However, between the two analyses, *Qinia hubeii* and its close relatives were shown to be more closely related to members of the Cymbellaceae (Fig 1), or the second major lineage of the Cymbellales that includes *Encyonema*, *Placoneis*, *Geissleria* and traditional members of the Gomphonemataceae. In each case, statistical support was high. The investigated strain CBac2022127, representing *Cymbella wuhanensis* sp. nov., is shown to form a monophyletic group with such *Cymbella* species as *C*. *aspera* (Ehrenberg) Cleve, *C*. *baicalaspera* Glushchenko, Kulikovskiy & Kociolek, *C*. *bengalensis* Grunow, and *C*. *himalaspera* Jüttner & Van de Vijver with high statistical supports (Fig 1: ML 100; BI 0.98; Fig 2: ML 100; BI 1.00). The phylogenetic relationships of the new species were sustained by genetic p-distance estimation, as all species presented higher genetic p-distances (0.05–0.23) with *Qinia hubeii* sp. nov. (strains CBac2022123 and CBac2022142) and *Cymbella wuhanensis* sp. nov. (strain CBac2022127). A minimal distance (0.05) observed between *Qinia hubeii* sp. nov. (strains CBac2022123 and CBac2022142) and strains CH021 and D170_008 of *Encyonopsis* sp. The p-distance between *Qinia hubeii* sp. nov. (strain CBac2022142) and *Cymbella wuhanensis* sp. nov. (strain CBac2022127) was 0.02 (data on genetic p-distance available from the authors).

### Species descriptions

#### *Qinia hubeii* Y. Liu & Kociolek sp. nov

Holotype: HANU. Slide no. THHB2022468, illustrated herein as Fig 4A.

Type strain: CBac2022123 and CBac2022142, isolated from samples THHB2022468 and THHB2022487 respectively. Deposited at Key laboratory of biodiversity of Aquatic Organisms, College of life science and technology, Harbin Normal University, China.

Isotype: COLO. Slide no. THHB2022468-1.

Type locality: Jiufeng reservoir, Wuhan city, Hubei Province.

Etymology: The specific epithet is chosen after the type locality.

Distribution: So far, the species is known only from its type locality.

**Fig 3:**
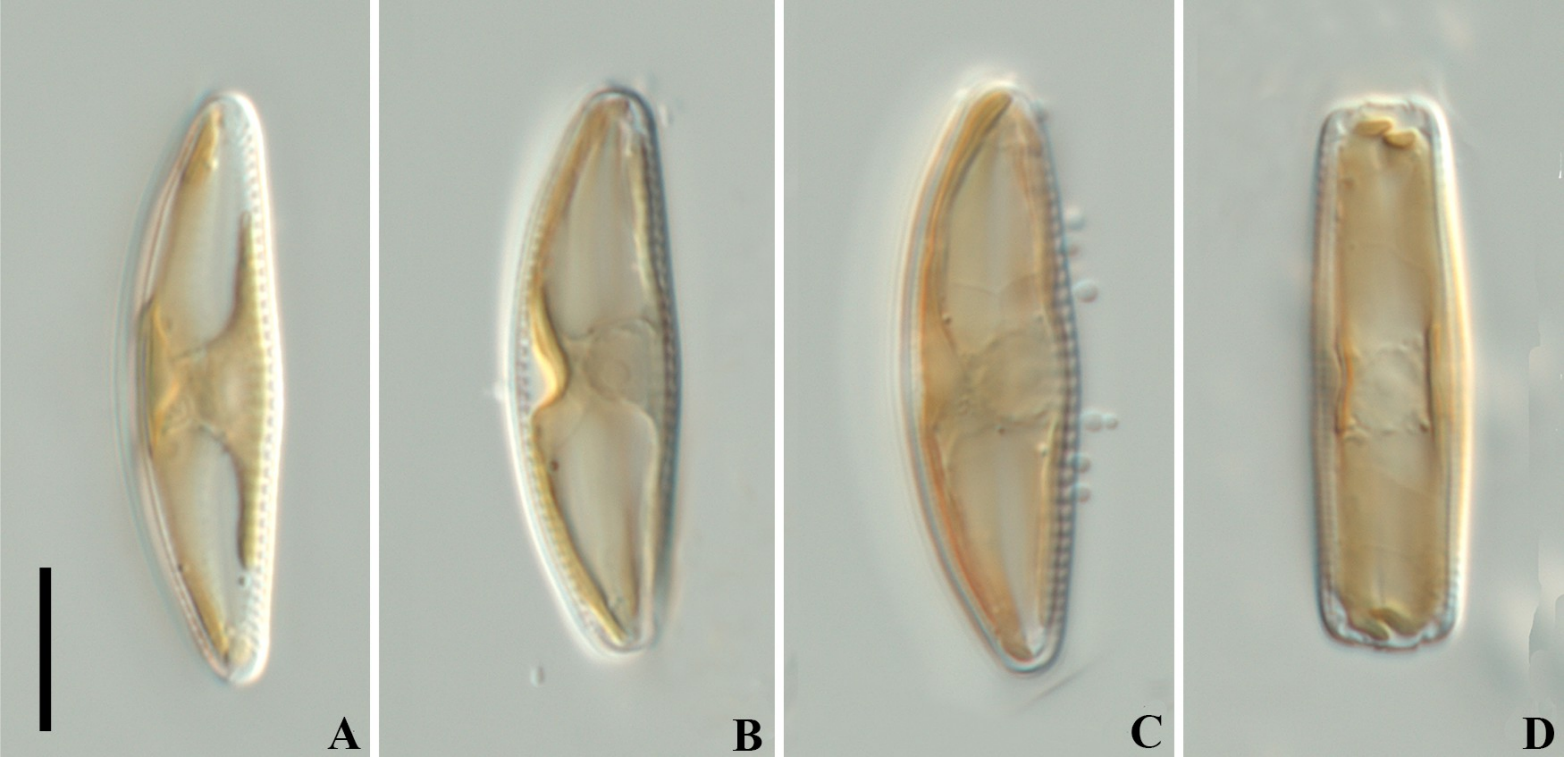
*Qinia hubeii* Y. Liu & Kociolek sp. nov., LM, differential interference contrast (DIC), live cells. Strain CBac2022123 and CBac2022142, slide No. CBac2022123 and CBac2022142. Morphology of protoplast, valve face view (A–C), girdle view (D). Scale bar = 10 μm.

Description:

Living cells (Fig 3): Cells solitary, rectangular in girdle view. Chloroplast H-shaped, close to the girdle, consisting of two plates connected by a wide isthmus.

**Fig 4:**
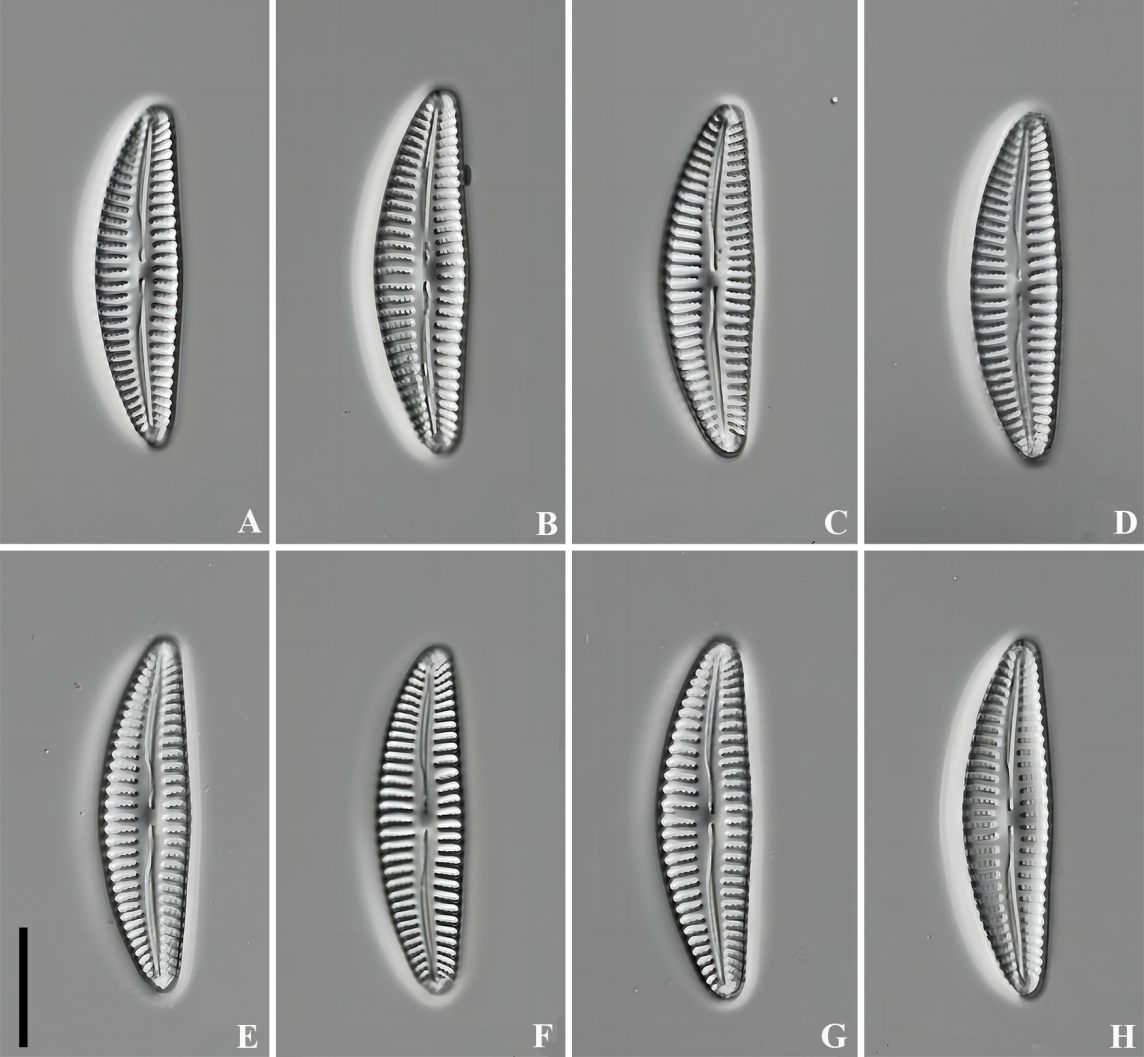
*Qinia hubeii* Y. Liu & Kociolek sp. nov., LM, (DIC), cleaned cells. Strain CBac2022123 and CBac2022142, slide No. CBac2022123 and CBac2022142. Size diminution series, including the holotype (A). Scale bar = 10 μm.

LM (Fig 4): Valve asymmetrical about the apical axis, with arched dorsal side, ventral side slightly tumid in the middle. Apices rounded, slightly acute, not protracted. Length 27.9–29.8 μm, breadth 6.4–7.9 μm. Raphe centrally positioned, axial area lanceolate, central area hardly expressed, almost as wide as axial area. Striae nearly parallel in the middle of the valve, becoming radiate near the apices, Striae density 10–11/10 μm in the middle, 13–14/10 μm near the apices. Areolae 16–18/10 μm.

**Fig 5:**
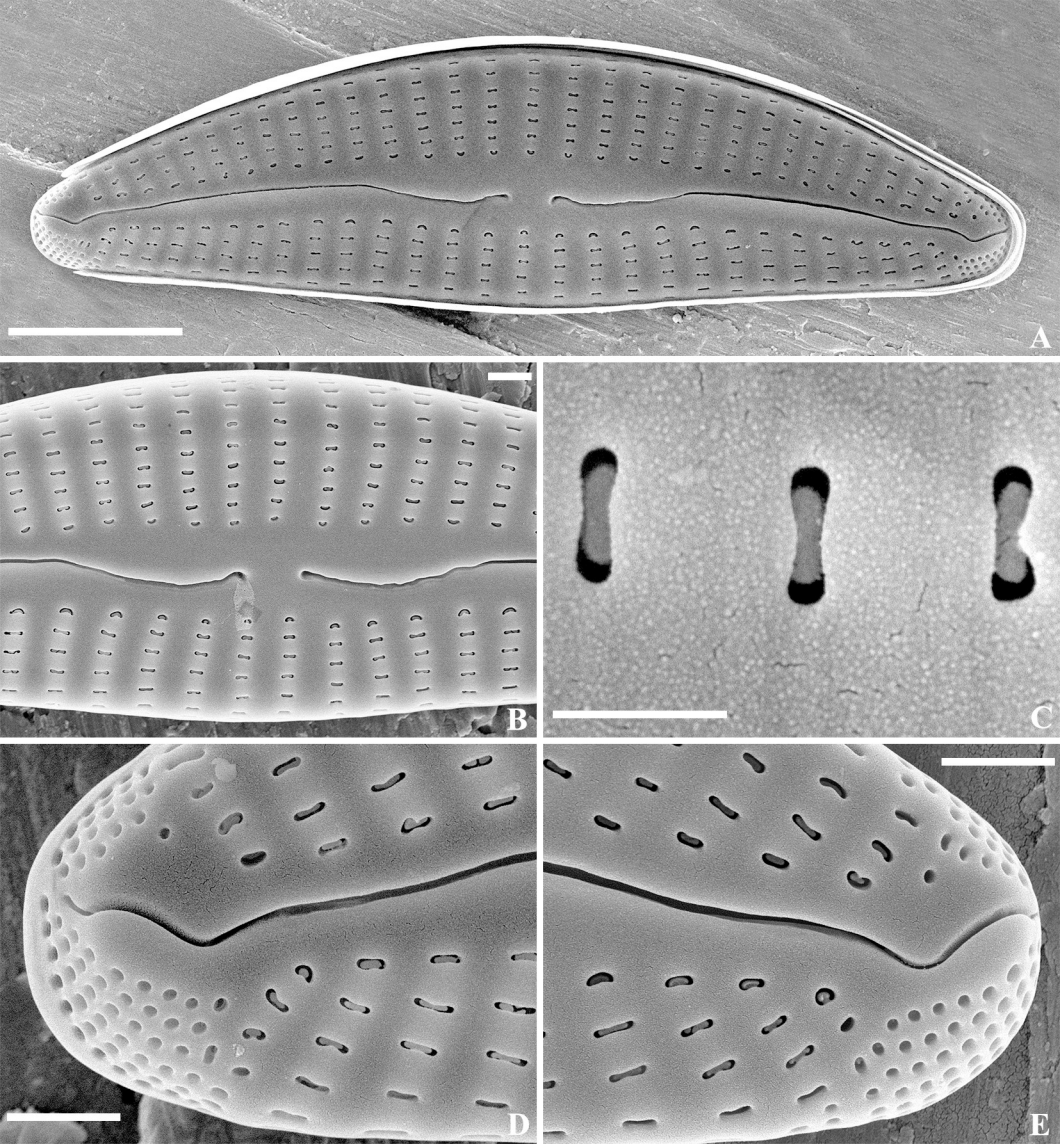
*Qinia hubeii* Y. Liu & Kociolek sp. nov., SEM, external view. Strain CBac2022123 and CBac2022142, slide No. CBac2022123 and CBac2022142. The whole valve (A). Details of the central area (B). Details of areolae structure (C). Details of the apices (D–E). Scale bars = 5 μm (A); 1 μm (B, D–E); 0.5 μm (Fig C).

SEM, external view (Fig 5): Raphe lateral, becoming reverse-lateral near the center, proximal raphe ends small and round, distal raphe ends bent to the dorsal side. APFs developed at both apices, to the mantle, porelli small and round, bisected by the raphe. Striae uniseriate, extending onto the mantle. Areolae predominantly apically elongated, slit-like and C-shaped along the axial area. APFs consisting of round porelli, unequally bisected by the raphe, dorsal portion smaller than ventral. Porelli differentiated from areolae in striae.

**Fig 6:**
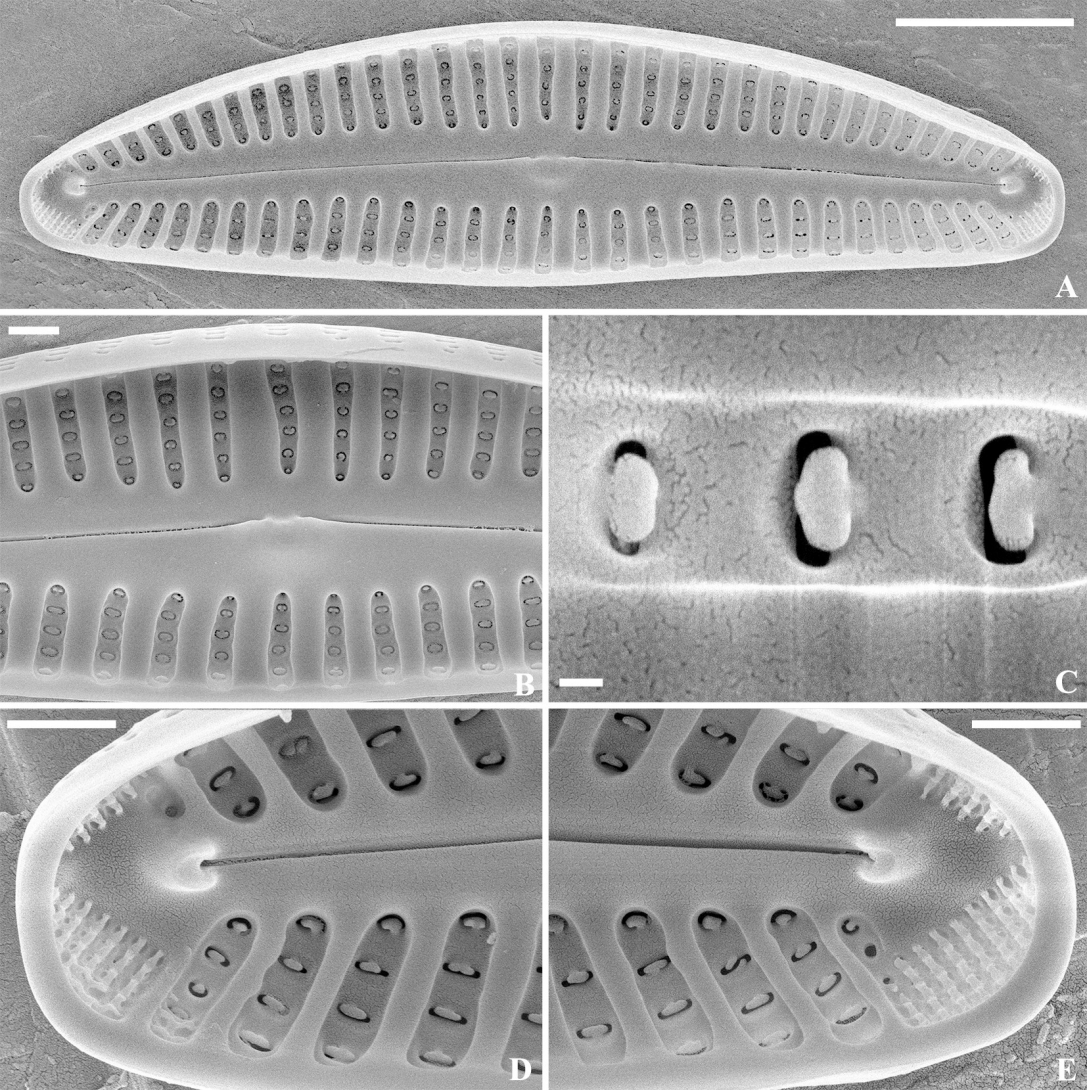
*Qinia hubeii* Y. Liu & Kociolek sp. nov., SEM, internal view. Strain CBac2022123 and CBac2022142, slide No. CBac2022123 and CBac2022142. The whole valve (A). Details of the central area (B). Details of areolae structure (C). Details of the apices (D–E). Scale bars = 5 μm (A); 1 μm (B, D–E); 0.1 μm (Fig C).

SEM, internal view (Fig 6). Raphe branches straight, proximal fissures covered with a narrow silica overgrowth (intermissio absent). Distal raphe ends terminate with small, slightly offset helictoglossae. Interstriae thickened. Areolae openings slit-like to C-shaped, partially covered by elliptical strutted occlusions. Parts of the APFs unequal, porelli positioned in rows, occluded.

*Cymbella wuhanensis Y*. *Liu & Kociolek* sp. nov.

Holotype: HANU. Slide no. THHB2022472, illustrated herein as Fig 8A.

Type strain: CBac2022127, isolated from sample THHB2022472. Deposited at Key laboratory of biodiversity of Aquatic Organisms, College of life science and technology, Harbin Normal University, China.

Isotype: COLO. Slide no. THHB2022472-1

Type locality: Jiufeng reservoir, Wuhan city, Hubei Province.

Etymology: The specific epithet is chosen after the type locality.

Distribution: So far, the species is known only from its type locality.

**Fig 7:**
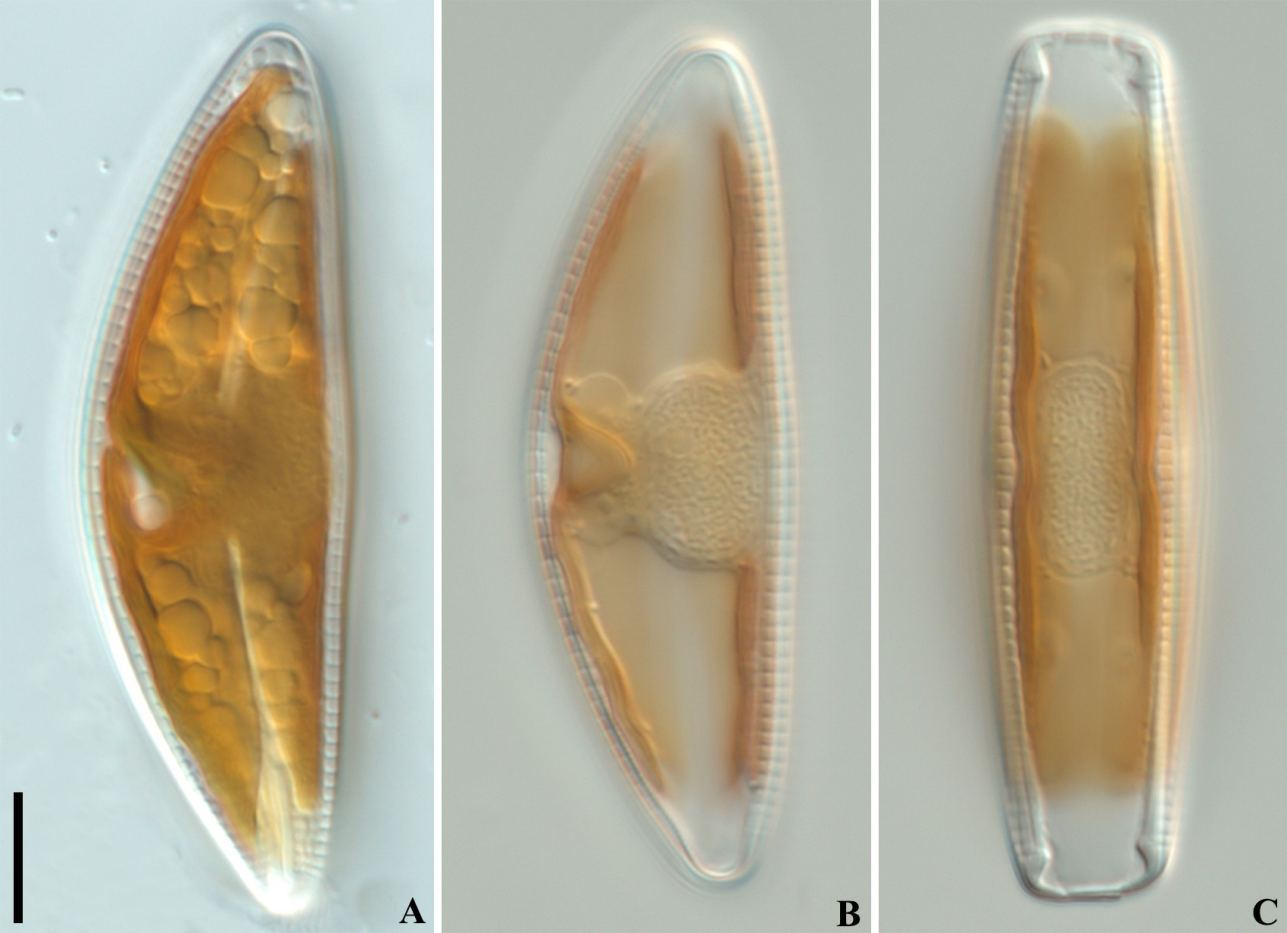
*Cymbella wuhanensis* Y. Liu & Kociolek sp. nov., LM, differential interference contrast (DIC), live cells. Strain CBac2022127, slide No. CBac2022127. Light microscopy, differential interference contrast. Morphology of protoplast, valve face view (A–B), girdle view (C). Scale bar = 10 μm.

Description:

Living cells (Fig 7): Cells solitary, rectangular in girdle view. Chloroplast H-shaped, close to the girdle, consisting with two plates connected by a wide isthmus.

**Fig 8:**
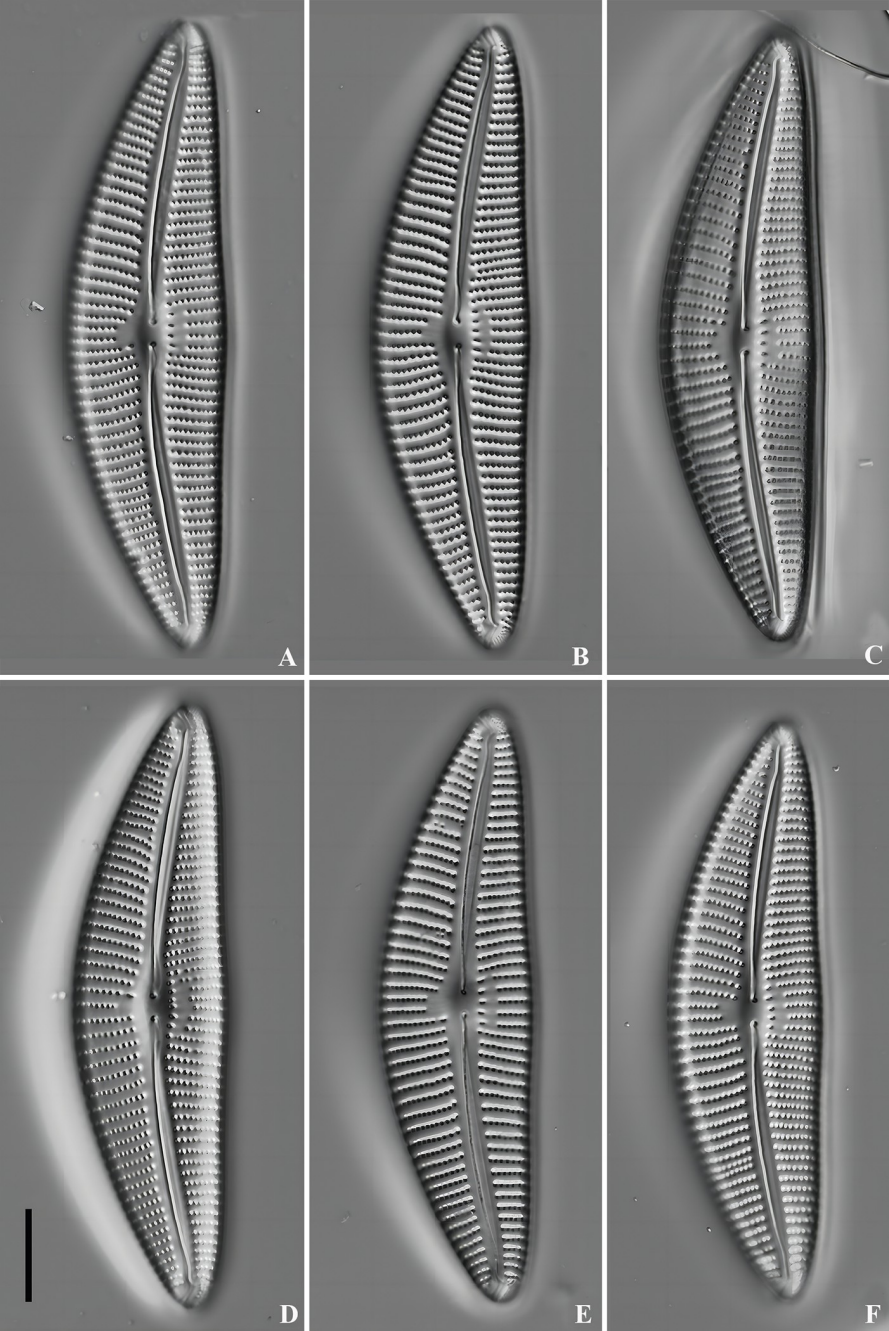
*Cymbella wuhanensis* Y. Liu & Kociolek sp. nov., LM, (DIC), cleaned cells. Strain CBac2022127, slide No. CBac2022127. Size diminution series, including the holotype (8A). Scale bar = 10 μm.

LM (Fig 8): Valve asymmetrical about the apical axis, with arched dorsal side, ventral side straight or slightly tumid in the middle, apices not protracted and narrowly rounded. Length 62.3–67.8 μm, breadth 16.1–16.9 μm. Raphe strongly lateral, narrowing towards the apices. Axial area linear and narrow, central area transversely elliptical, with 5–7 ventral stigmata. Striae nearly parallel in the middle, becoming radiate near the apices. Striae 8–9/10 μm in the middle, 9–11/10 μm near the apices. Areolae 14–15/10 μm.

**Fig 9:**
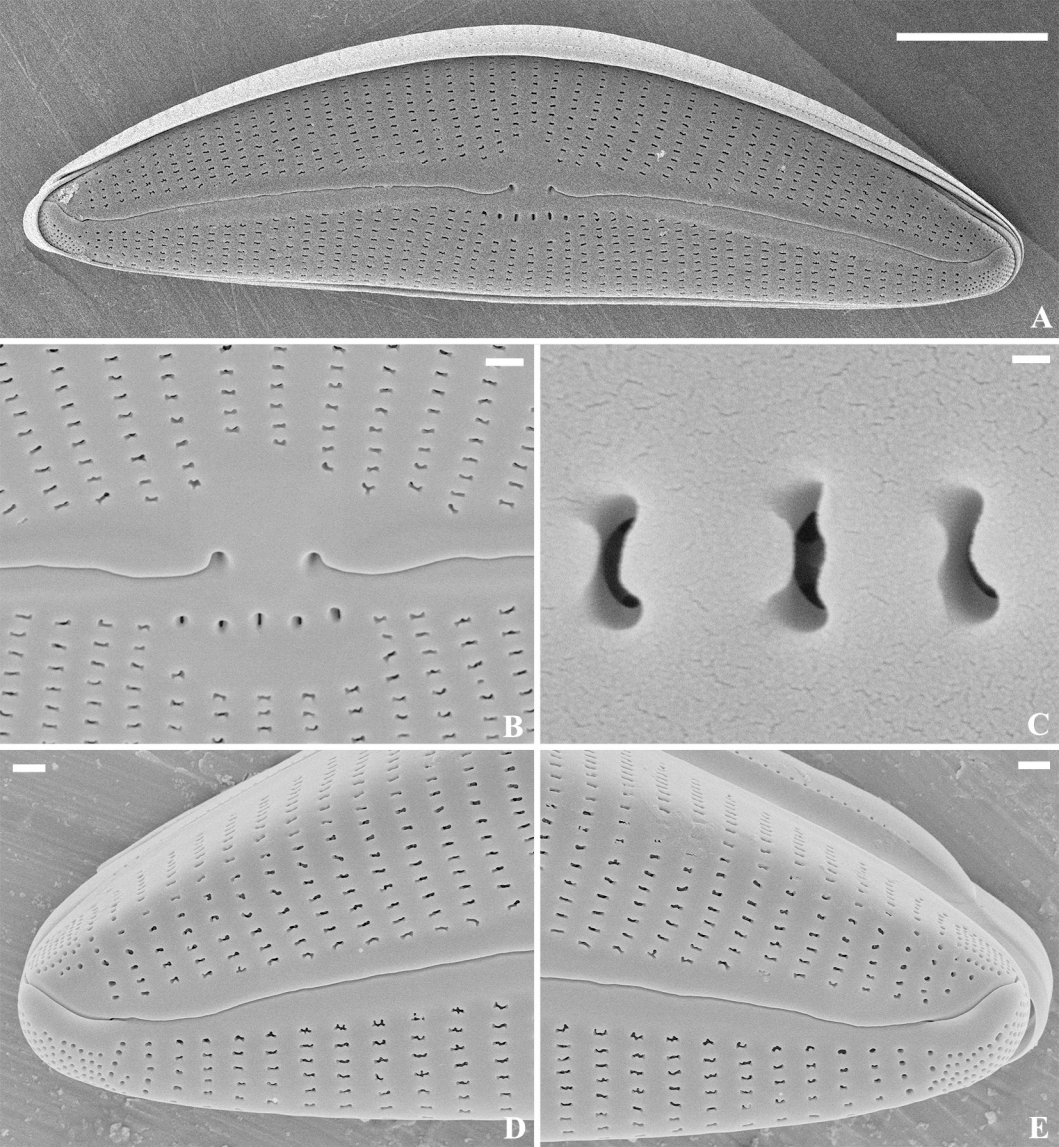
*Cymbella wuhanensis* Y. Liu & Kociolek sp. nov., SEM, external view. Strain CBac2022127, slide No. CBac2022127. The whole valve (A). Details of the central area (B). Details of areolae structure (C). Details of the apices (D–E). Scale bars = 5 μm (A); 1 μm (B, D–E); 0.5 μm (C).

SEM, external view (Fig 9): Raphe lateral, slightly reverse in the central valve portion, proximal raphe ends small and round, distal raphe ends bent to the dorsal side. Striae uniseriate. Areolae apically-elongated, slit-like, sometimes slightly irregular shaped, with jagged sides, extend onto the mantle. Very small unilateral projections are often present. Ventral side of the central area with 5 stigmata with transverse slit-like openings, isolated from striae. APFs present at both apices, bisected by the distal raphe fissures into dorsal and ventral side, extend to the mantle. Porelli small and round, similar to areolae of the adjacent striae in shape and size.

**Fig 10:**
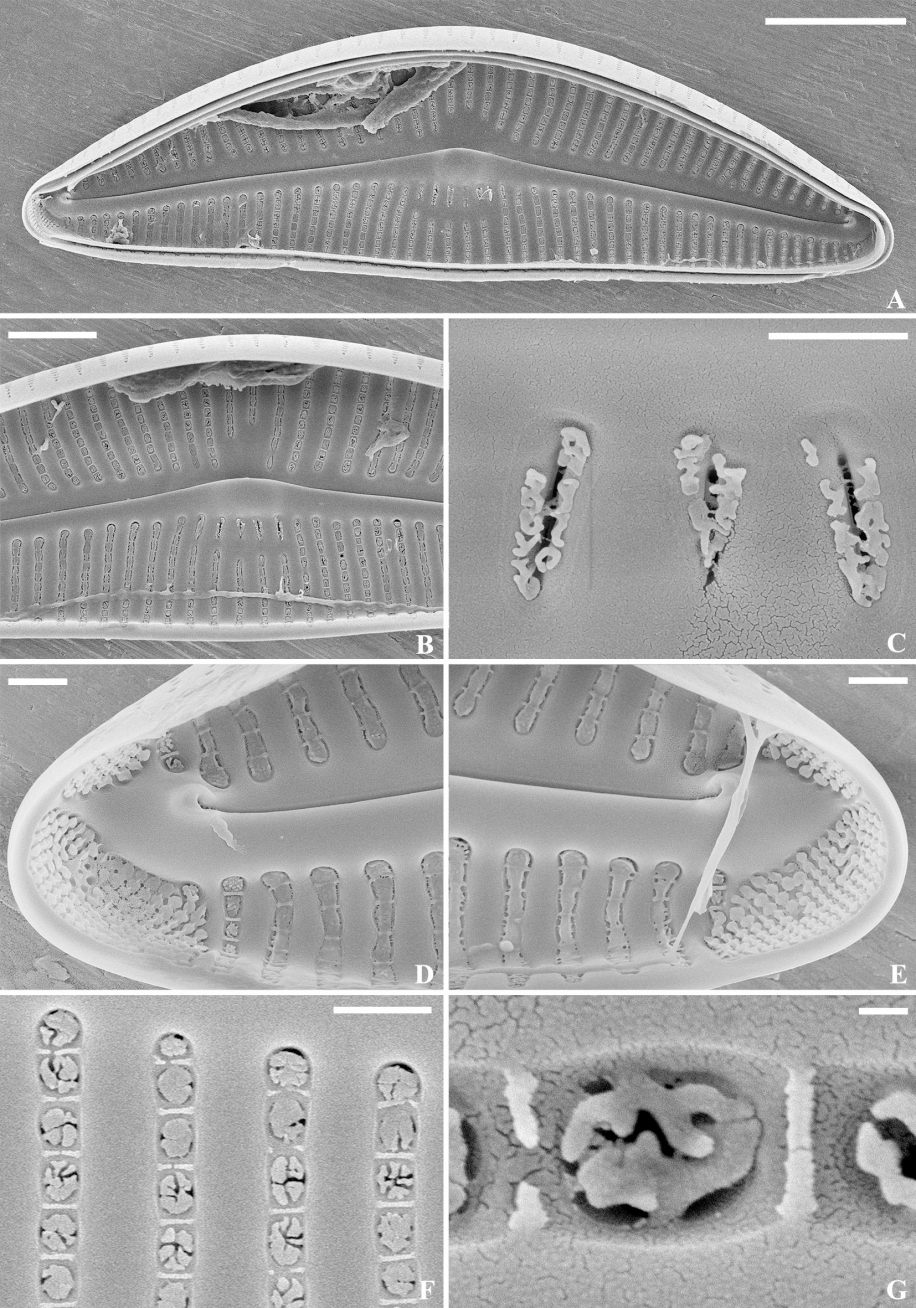
*Cymbella wuhanensis* Y. Liu & Kociolek sp. nov., SEM, internal view. Strain CBac2022127, slide No. CBac2022127. The whole valve (A). Details of the central area (B). Details of the stigma structure (C). Details of the apices (D–E). Details of areolae structure (F–G). Scale bars = 10 μm (A); 5 μm (B); 1 μm (C–F); 0.1 μm (G).

SEM, internal view (Fig 10): Raphe branches straight, proximal fissures covered with a silica overgrowth (intermissio absent). Distal raphe ends terminate with small, slightly offset helictoglossae. Interstriae slightly thickened. Areolae openings covered with flaps, similar with the structure of ’pseudotectulum’ (see Mironov et al. [25]). Internal openings of stigmata slit-like, with irregular outgrowths. APFs bisected into unequal parts, porelli positioned in rows, occluded.

## Discussion

### Phylogenetic relationships of genera within Cymbellales

While the Cymbellales has been long thought to represent a natural group of taxa [26–28], morphological and molecular approaches to understanding relationships within the group have yielded many alternative hypotheses. Some have suggested relationships based on symmetry, recognizing divisions between cymbelloid diatoms (those with asymmetrical to the axis) and gomphonemoid diatoms (those with asymmetrical about the transapical axis) [27,29–30]. Kociolek and Stoermer [31] showed that *Didymosphenia* did not follow that organization, since even though it has gomphonemoid symmetry, its valve ultrastructure and cytoplasmic features are more similar to cymbelloid diatoms than gomphonemoid diatoms (a finding supported in numerous molecular studies, e.g. Nakov et al. [32] and herein). Bruder and Medlin [33] showed with molecular data that naviculoid diatoms such as *Placoneis* and *Geissleria* should be included within this group. However, there have been few similarities between analyses considering the composition of the major lineages within the Cymbellales and even the monophyly of genera [32–39]. Even in the analyses presented herein, there are discrepancies between the two approaches (more taxa with fewer sequences versus fewer taxa with longer sequences) with respect to hypotheses of evolutionary descent. Clearly, further work on the Cymbellales is necessary to untangle relationships of the group.

### On the phylogeny of *Qinia* and *Cymbella wuhanensis* sp. nov. and morphology of the new species

*Qinia* has been recently discovered in China and described as a new genus based on a unique combination of morphological features–astigmate valves, APFs unequally bisected by the distal raphe fissures, areolae with slit-like external openings and unilateral foricula occluding the areolar openings internally [13]. The newly introduced taxon *Qinia hubeii* sp. nov. demonstrates the same combination of valve features. In this study, molecular analysis upholds the phylogenetic position of *Qinia* in the order Cymbellales. And, as our data strongly supports, two strains of *Q*. *hubeii* sp. nov. represent an independent genus, *Qinia*, which is separated from *Cymbella*. Another species described in this paper, *Cymbella wuhanensis* sp. nov., is characterized by possessing an interesting suite of morphological features. In some regards it resembles species of the *Cymbella proxima*-group, studied by Krammer [4]. Taxa within this group are united by numerous morphological features: “cistuloid” outlines, reverse-lateral raphes, orbicular central areas and several stigmata, isolated from the striae. These characters are also found in *C*. *wuhanensis* sp. nov. Morphologically, the new species is most similar to *Cymbella sinensis* Krammer, which is a unique representative of the genus *Cymbella*: in this species, APFs are unequally bisected by the raphe fissures, areolar openings are jagged externally, with small strutted projections ([4]: pl. 121, Figs 5–8). Thus, both *C*. *wuhanensis* sp. nov. and *C*. *sinensis* are similar to *Qinia* in structure of the APFs. In the constructed trees, *C*. *wuhanensis* sp. nov. and *Qinia hubeii* sp. nov. are separated from each other and belong to separate clades. Thisdistance in the phylogenetic trees between suggests that this APF condition is homoplasous, having evolved independently in these taxa (and, separately, in *Reimeria*; [40]). However, the two *Cymbella* species differ from *Qinia* by valve symmetry and outline: *C*. *wuhanensis* sp. nov. and *C*. *sinensis* have large valves with strongly arched dorsal margin and straight to slightly tumid ventral margin, areolae with jagged external openings and small projections. In *Qinia*, the valves are smaller, with more acute apices, areolae slit-like externally, with unilateral foricula internally. In addition, *C*. *wuhanensis* sp. nov. has 5–7 stigmata, while in *Qinia* stigmata are absent [13]. The major evidence for the taxonomic position of *C*. *wuhanensis* sp. nov. in *Cymbella* s.s. is molecular data, acquired during the current study. The investigated strain of *C*. *wuhanensis* sp. nov. forms a monophyletic group with the species of *Cymbella aspera*-group with high statistical supports. This incongruence between morphological and molecular data argues for continued study on the Cymbellales, likely requiring additional taxon sampling, molecular markers and morphological analyses. Morphological characters of *Qinia hubeii* sp. nov., *Cymbella wuhanensis* sp. nov. and similar taxa are compared in Tables 2 and 3.

**Table 2:** Morphological comparison of *Qinia hubeii* sp. nov. with similar taxa.

Taxon	*Q*. *hubeii* sp. nov.	*Q*. *lashii*	*Q*. *aequalis*
**Length (μm)**	27.9–29.8	37–51	21.3–29.0
**Width (μm)**	6.4–7.9	9–11	7.5–9.6
**Stria in 10 μm**	10–11 mid-valve, 13–14 near the apices	7–9	7–9
**Areolae in 10 μm**	16–18	21–22*	25–27*
**Valve outline**	Arched dorsally, slightly tumid ventrally	Arched dorsally, slightly concave ventrally, slightly tumid mid-valve	Lanceolate-elliptic, moderately dorsiventral
**Valve ends**	Not protracted, rounded, slightly acute	Not protracted, acutely rounded	Not protracted, acutely rounded
**Raphe**	Mostly lateral, reverse-lateral in middle of the valve	Lateral throughout the valve	Lateral throughout the valve, undulate
**Striae**	Parallel mid-valve, radiate near the apices	Parallel to slightly radiate mid-valve, radiate near the apices	Parallel to slightly radiate mid-valve, radiate near the apices
**Stigmata**	0	0	0
**Apical pore fields**	Unequally bisected, consisting of round differentiated porelli	Unequally bisected, consisting of round differentiated porelli	Unequally bisected, consisting of round differentiated porelli
**Reference**	This study	[13]	[13]

* counted from published data.

**Table 3:** Morphological comparison of *Cymbella wuhanensis* sp. nov. with similar taxa.

Taxon	*C*. *wuhanensis* sp. nov.	*C*. *sinensis*	*C*. *proxima*	*C*. *kemiana*	*C*. *amplificata*	*C*. *baicalensis*
**Length (μm)**	62.3–67.8	48–58	38–120	45–74	75–160	112–195
**Width (μm)**	16.1–16.9	17–28	18–24	17–21	20–31	51–60
**Stria in 10 μm**	8–9 mid-valve, 9–11 near the apices	12–13	7–10 mid-valve, ca. 11/10 μm near the apices	9–10 mid-valve, ca. 12/10 μm near the apices	7–13	6–7
**Areolae in 10 μm**	14–15	15–16	12–18	20–23	12–17	ca. 8
**Valve outline**	Arched dorsally, straight or slightly tumid centrally	Strongly arched dorsally, straight or slightly concave ventrally, slightly tumid mid-valve	Strongly arched dorsally, moderately concave ventrally	Strongly arched dorsally, straight or slightly concave ventrally, tumid mid-valve	Moderately arched dorsally, straight to slightly concave ventrally, slightly tumid mid-valve	Strongly arched dorsally, straight or slightly concave ventrally, slightly tumid mid-valve
**Valve ends**	Not protracted, narrowly rounded	Not protracted, narrowly rounded	Not protracted, broadly rounded or acuminate	Not protracted, narrowly rounded	Not protracted, broadly rounded	Not protracted, narrowly rounded
**Raphe**	Strongly lateral, narrowing towards the apices	Moderately lateral, filiform towards the apices and near the proximal ends	Lateral, filiform near the apices	Slightly lateral, filiform towards the apices, reverse-lateral near the proximal ends	Lateral, filiform towards the apices and near the proximal ends	Strongly lateral, filiform towards the apices and near the proximal ends
**Striae**	Parallel mid-valve, radiate near the apices	Radiate throughout the valve	Radiate throughout the valve	Slightly radiate mid-valve, more radiate at the apices	Radiate throughout the valve	Radiate throughout the valve
**Stigmata**	5	0	2–5	2–4	>7	4–6
**Apical pore fields**	Unequally bisected, porelli, similar to areolae in adjacent striae	Unequally bisected, porelli, differentiated from areolae in striae	Not bisected, porelli differentiated from areolae in striae	Present, structure unknown	Present, structure unknown	Present, structure unknown
**Reference**	This study	[4]	[4]	[4]	[4]	[4]

*Qinia hubeii* sp. nov. is endemic of China and differs from *Qinia lashii* Y. Liu & Kociolek by a number of morphological features, such as smaller valves (length 27.9–29.8 vs 37.0–51.0 in *Q*. *lashii*; breadth 6.4–7.9 vs 9.0–11.0 in *Q*. *lashii*) with less acute, not protracted apices and more narrow axial area ([13]: Fig 2). *Qinia aequalis* Liu & Kociolek is most similar to *Q*. *hubeii* sp. nov. Both species are characterized by lanceolate-elliptic valve outlines, lateral raphe, linear axial and central areas. However, in *Q*. *aequalis* valves are shorter (21.3–29.0 vs 27.9–29.8 in *Q*. *hubeii* sp. nov.), but wider (7.5–9.6 vs 6.4–7.9 in *Q*. *hubeii* sp. nov.), striae are sparser (7–9 vs 10–11 in 10 μm in *Q*. *hubeii* sp. nov.) ([13]: Fig 5). Based on its valve morphology, *Cymbella wuhanensis* sp. nov. could be allocated within the genus *Qinia*, but the molecular data showed very different results, allying it with *Cymbella*. Although the outlines are very similar with *Cymbella*, the valve structure is, in fact, very distinctive. In *C*. *wuhanensis* the APFs are bisected by the raphe which is a typical feature in *Qinia*. Among the species of *Cymbella*, those equipped with stigma possess 1–7 of these structures. Stigmata are located ventrally, beside the central area, separated from areolae in striae, with openings internally covered by teeth-like structures or surrounded by rugged margins ([4], p. 200, plate 5). *Cymbella wuhanensis* sp. nov. has 5–7 stigma in the central area, with small round external opening like most typical *Cymbella*, but the internal opening was with very irregular overgrowths around the margin of the alveoli, which is a unique feature within the genus. The areolae are mostly unoccluded in *Cymbella*, e.g. in *Cymbella hechiensis* Li & W. Zhang [41], *Cymbella neocistula* [4] etc. Several species are characterized by having areolae with round or ellipical papillae on the inside ([4]: plates 60, 79, 104). However, in *C*. *wuhanensis* sp. nov. areolae are equipped with irregularly structured occlusions, similar to pseudotectula, found in *Witkowskia* [25], which is quite unusual, too. Based on the molecular data, *C*. *wuhanensis* sp. nov. seems to be very closely related to *Cymbella aspera*, as they were clustered together in both trees, but the species do not share similarities in valve structures and are easily separated by a set of morphological characters. The morphology of *Cymbella wuhanensis* sp. nov. and representatives of *Cymbella proxima*-group is very similar. For instance, the structure of stigmata and striae is identical between the new species and *C*. *proxima* ([4]: pl. 113, Figs 2–4). However, in *C*. *wuhanensis* sp. nov., the valve ends are more acute, less protracted and the ventral margin less tumid. The same features can be used to distinguish *C*. *wuhanensis* sp. nov. from *Cymbella kemiana* Krammer ([4]: pl. 115, Figs 1–7). *Cymbella amplificata* Krammer differs from the new species by having broader apices, more rounded central area and greater number of stigmata (>7 vs 5–7 in *C*. *wuhanensis* sp. nov.) ([4]: pl. 118, Figs 1–3; pl. 119, Figs 1–5). From *Cymbella baicalensis* Skvortzov & Meyer the new species can be distinguished by much smaller valves (length 112.0–195.0 vs 62.3–67.8 in *C*. *wuhanensis*; breadth 51.0–60.0 vs 16.1–16.9 in *C*. *wuhanensis*), a less orbicular central area and more prominent stigmata ([4]: pl. 120, Figs 1–4). Among the species of *Cymbella proxima*-group, *Cymbella sinensis* is most similar to *C*. *wuhanensis*. Moreover, both species were described from China. Despite the similarities in APFs and areolae structure, *C*. *sinensis* can be distinguished by having broader apices, smaller central area and absence of stigmata. Additionally, distal fissures of the raphe are gradually curved in *C*. *wuhanensis* sp. nov., but abruptly bent in *C*. *sinensis* ([4]: pl. 121, Figs 1–8).

## Supporting information

S1 Appendix Alignment of the *rbcL* and *18S rRNA* genes used for phylogenetic analyses in this study. (TXT)

S2 Appendix The Bayesian phylogenetic topology for the *rbcL* and *18S rRNA* genes tree. (TXT)

S3 Appendix Alignment of the *rbcL*, *18S rRNA* and *28S rRNA* genes used for phylogenetic analyses in this study. (TXT)

S4 Appendix The Bayesian phylogenetic topology for the *rbcL*, *18S rRNA* and *28S rRNA* genes tree. (TXT)
